# Germline pathogenic variants in *RNF43* in patients with and without serrated polyposis syndrome

**DOI:** 10.1007/s10689-024-00428-6

**Published:** 2024-11-15

**Authors:** Heidi Hesselø Brinch, Anna Byrjalsen, Zuzana Lohse, Andreas Ørslev Rasmussen, John Gásdal Karstensen, Britta Schlott Kristiansen, Anne Marie Jelsig

**Affiliations:** 1 Department of Clinical Genetics, Copenhagen University Hospital, Copenhagen, Denmark; 2 Department of Clinical Genetics, Zealand University Hospital, Roskilde, Denmark; 3 Department of Genomic Medicine, Copenhagen University Hospital, Copenhagen, Denmark; 4 Danish Polyposis Register, Gastro Unit, Copenhagen University Hospital – Amager and Hvidovre and Department of Clinical Medicine, University of Copenhagen, Copenhagen, Denmark; 5 Department of Clinical Genetics, Odense University Hospital, Odense, Denmark

**Keywords:** Colorectal cancer, RNF43, Serrated polyposis syndrome, Polyposis, Cancer predisposition syndrome, Cancer genetics

## Abstract

Serrated Polyposis Syndrome (SPS) is characterized by multiple and/or large serrated polyps in the colon and an increased risk of colorectal cancer (CRC). The etiology is largely unknown, but in a subset of patients with SPS, monoallelic pathogenic variants in *RNF43* are detected. To date, however, the penetrance and phenotypic spectrum of patients carrying pathogenic variants (PV) in *RNF43* are poorly described. We present eight patients both with and without serrated polyps from four unrelated families with likely pathogenic variants (LPV) in *RNF43* and compare the results to current literature. The patients were referred to genetic counseling due to suspicion of hereditary cancer. They underwent genetic testing with custom NGS gene panels including *RNF43* as part of a routine genetic work-up. Three LPVs, one multi-exon deletion and two nonsense variants, were detected in four families. Family I had a history of CRC and serrated polyps, but in the three other families (II‒IV) there was no history of CRC or serrated polyps. Colonoscopies in the probands of these families did not reveal any serrated polyps and/or CRC despite some of them being relatively old. Our findings suggest that the penetrance of *RNF43*-related disease is much lower than previously thought, and raise questions about the connection between *RNF43* and disease. The results highlight the complexity of genetic counseling in *RNF43* positive families– particularly in families without polyposis. Further research is needed to elucidate the role of *RNF43* in the risk of SPS and CRC.

**Supplementary Information:**

The online version contains supplementary material available at 10.1007/s10689-024-00428-6.

## Introduction

Serrated Polyposis Syndrome (SPS) is characterized by multiple colorectal serrated polyps and an increased risk of colorectal cancer (CRC). The prevalence is unknown. In cohorts screened for fecal occult blood subsequent colonoscopies found SPS in 1:111‒1:238 individuals [1, 2], making SPS the most common polyposis syndrome. According to the WHO criteria (2019) the SPS diagnosis can be allotted if fulfilling at least one of the following:

- ≥ 5 serrated lesions/polyps proximal to the rectum, all being ≥ 5 mm in size, with at least 2 polyps ≥ 10 mm in diameter.
- 20 serrated lesions/polyps of any size distributed throughout the large bowel, with at least ≥ 5 proximal to the rectum [3].

Although first and second-degree relatives of patients with SPS seem to be at an increased risk of CRC [4, 5], the condition does not appear to follow classic Mendelian inheritance. The etiology is largely unknown, however, association to various risk factors (female sex, smoking) has been identified [6]. In 2014, *RNF43* was first linked to SPS [7]. In a small subset of patients with SPS monoallelic pathogenic variants (PVs) in *RNF43* have been detected and regarded causative [8–10] To the authors knowledge, ten families with PVs in *RNF43* have been reported in the literature (Table 1), thus little is known about the phenotypic spectrum, penetrance, and interfamilial variability. In this short report we present four novel families with likely pathogenic variants (LPVs) in *RNF43*, explore clinical phenotypes and segregation, thus adding to our knowledge of *RNF43* related disease. And we raise the question: is *RNF43* a monogenetic cause of SPS and can it be applied in broad cancer gene panels?

**Table 1:** Phenotypic spectrum of patients carrying a pathogenic variant in *RNF43*

Publication	Family and patient	RNF43 variant (NM_017763.6, NP_060233.3)	Sessile serrated polyps	Age at first serrated polyp detected	Other types of polyps	Fulfilling the WHO Criteria (2019)	Personal history of cancer (age)	Family history of serrated polyps (FDR, SDR)	Family history of colorectal cancer (FDR, SDR)
Brinch et al. (this study)	Family 1, patient 1	c.375 + 1_376-1)_(2308 + 1_2309-1)del	> 20	46	Yes	Yes	No	Yes (SDR)	Yes (FDR, SDR)
	Family 1, patient 2	c.375 + 1_376-1)_(2308 + 1_2309-1)del	No	N/A	N/A	No	CRC (64)	Yes (FDR)	Yes (FDR)
	Family 1, patient 3	c.375 + 1_376-1)_(2308 + 1_2309-1)del	5–20	61	Yes	N/A	CRC (70)	Yes (SDR)	Yes (FDR, SDR)
	Family 1, patient 4	c.375 + 1_376-1)_(2308 + 1_2309-1)del	N/A	N/A	N/A	N/A	No	Yes (FDR)	Yes (SDR)
	Family 1, patient 5	c.375 + 1_376-1)_(2308 + 1_2309-1)del	N/A	N/A	N/A	N/A	No	Yes (FDR)	Yes (SDR)
	Family 2, patient 1	c.1948 C > T, p.(Arg650Ter)	No	-	Yes	No	GC (61)	No	No
	Family 3, patient 1	c.1948 C > T, p.(Arg650Ter)	N/A	N/A	N/A	No	No	N/A	N/A
	Family 4, patient 1	c.1009 C > T, p.(Arg337Ter)	N/A	N/A	N/A	No	No	No	No
Gala et al. [7]	Family 1, Patient 1	c.337 C > T, p.(Arg113Ter)	> 20	51	N/A	Yes	No	N/A	Yes (FDR)
	Family 2, Patient 1	c.337 C > T, p.(Arg113Ter)	5–20	52	N/A	N/A	CLL (42)	N/A	Yes (SDR)
Taupin et al. [9]	Family 1, patient 1	c.394 C > T, p.(Arg132Ter)	”Multiple polyps”	23	N/A	Yes	CRC (23)	Yes (FDR)	No
	Family 1, patient 2	c.394 C > T, p.(Arg132Ter)	> 20	27	No	Yes	No	Yes (FDR)	Yes (FDR)
Buchanan et al. [10]	Family 1, Patient 1	c.640 C > G, p.(Leu214Val)	> 20	18	N/A	Yes	N/A	N/A	N/A
	Family 2, Patient 1	c.443 C > G, p.(Ala148Gly)	> 20	57	N/A	Yes	N/A	N/A	N/A
Yan et al. [8]	Family 1, patient 1	c.953-1G > A, p.(Glu318Glyfs*124)	> 20	65	Yes	Yes	No	Yes (FDR)	Yes (FDR)
	Family 1, Patient 2	c.953-1G > A, p.(Glu318Glyfs*124)	> 20	64	N/A	Yes	No	Yes (FDR)	Yes (FDR)
	Family 1, Patient 3	c.953-1G > A, p.(Glu318Glyfs*124)	*≤* 5	49	N/A	No	CRC (49)	Yes (FDR)	No
	Family 1, Patient 4	c.953-1G > A, p.(Glu318Glyfs*124)	”several polyps”	37	N/A	Yes	No	Yes (FDR)	Yes (FDR)
	Family 1, Patient 5	c.953-1G > A, p.(Glu318Glyfs*124)	”several polyps”	35	N/A	Yes	No	Yes (FDR)	Yes (FDR)
	Family 1, Patient 6	c.953-1G > A, p.(Glu318Glyfs*124)	N/A	-	N/A	No	No	Yes (FDR)	Yes (FDR)
Quintana et al. [11]	Family 1, Patient 1	c.394 C > T, p.(Arg132Ter)	> 20	55	Yes	Yes	CRC (55)	Yes (FDR)	Unknown
Mikaeel et al. [12]	Family 1, Patient 1	c.375 + 1G > A, p.(Ala126Ilefs*50)	N/A	-	Yes	No	CRC (50)	Yes (FDR)	Yes (FDR)
	Family 1, Patient 2	c.375 + 1G > A, p.(Ala126Ilefs*50)	*< 5*	65	Yes	N/A	CRC (65)	No	Yes (FDR)

**Abbreviations**: CRC = Colorectal cancer, CLL = Chronic Leukocytic Leukemia, FDR = First degree relative, GC = Gastric Cancer, N/A = not available, SDR = Second degree relative

## Methods

Patients were referred for genetic counseling on suspicion of hereditary cancer. Genetic testing was performed on DNA extracted from peripheral blood. A customized NGS gene panel contained at least the following genes: *APC*,* AXIN2*,* BMPR1A*,* EPCAM*,* GREM1*,* MLH1*,* MSH2*,* MSH3*,* MSH6*,* MUTYH*,* NTHL1*,* PMS2*,* POLD1*,* POLE*,* PTEN*,* SMAD4*,* STK11*, and *RNF43.* The latest version of the gene panel was a custom Twist Bioscience capture, sequenced on Illumina NextSeq550. Reads were mapped to the human reference genome GRCh38, and single nucleotide variants (SNVs) (Variant allele frequency > 20%) and copy number variations (CNVs) were called with GATK HaplotypeCaller and GermlineCNVcaller or Dragen/VarSeqCNV, respectively. Variant annotation and filtration were performed in VarSeq (Golden Helix, Bozeman MT, USA). In case of deceased patients/relatives, the analyses were performed on either DNA from a blood sample collected before death or from formalin-fixed, paraffin-embedded non-neoplastic tissue from an affected relative. The sequencing analysis enabled detection of SNVs in the coding regions and +/- 20–50 bp of the surrounding intronic regions, and detection of CNVs. We used non-gene specific ACMG guidelines for variant classification, which we acknowledge, require that the gene and disease in question follow Mendelian inheritance.

## Results

### Clinical Data

#### Family I (Fig. 1)

**Fig. 1:**
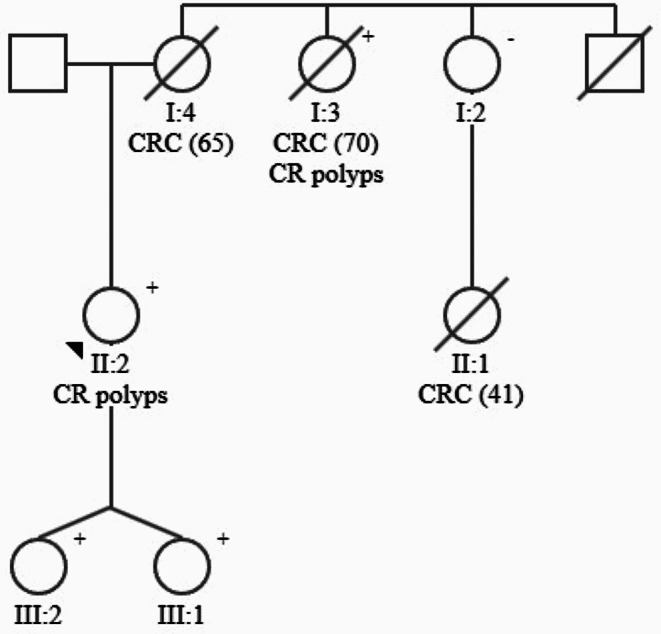
Pedigree of family 1 with colorectal cancer and sessile serrated polyps. We have opted against displaying pedigrees for families II, III and IV, as no segregation analysis of the RNF43 variants or further genetic work-up were performed in these families because of the absence of phenotypic SPS and no known history of CRC among the probands or their relatives

The proband (II:2), a 56-year-old Caucasian woman, underwent genetic counseling as her mother (I:4), an aunt (I:3) and a maternal cousin (II:1) all developed CRC. IHC showed normal expression for I:3 and II:1, whereas I:4 lacked expression of MLH1 and PMS2. Prior to genetic counselling, the patient (II:2) had undergone colonoscopies every 3 years since the age of 44 and 6 tubular adenomas and 42 sessile serrated polyps had been removed. The mother was diagnosed with CRC (mucinous adenocarcinoma) at age 65. The aunt was diagnosed with CRC in cecum and the ascending colon (adenocarcinoma) at age 70. The aunt had previously had 13 sessile serrated polyps (2 were > 20 mm) and 3 tubular adenomas removed. The maternal cousin was diagnosed with a CRC (adenocarcinoma) located in the hepatic flexure at age 41 (See Fig. 1).

Germline genetic testing was performed in the proband. The patient carried a germline heterozygous deletion of exon 4‒9 in *RNF43* (c.375 + 1_376-1)_(2308 + 1_2309-1)del) not previously reported in gnomAD. The variant was classified as likely pathogenic (class 4) according to ACMG guidelines. Direct gene test for the variant was performed on DNA from previously collected blood from the maternal aunt (I:3). Following, I:4 was an obligate carrier. Large deletions can be difficult to identify in FFPE tissue, thus tissue from the deceased maternal cousin could not reliably be used. Instead, the mother of the maternal cousin (I:2) was tested for the variant, which she did not carry. The variant has recently been detected in the proband’s twin daughters (III:1 and III:2, zygote unknown). None of the daughters have undergone endo- or colonoscopy making their polyp status unknown.

#### Family II

A 61-year-old Asian woman, underwent genetic counseling due to gastric cancer (adenocarcinoma) at age 61. IHC showed loss of MSH2 and MSH6 expression and normal expression of MLH1 and PMS2. Four years prior to the diagnosis, the patient had a colonoscopy which revealed a single tubular adenoma.

**Fig. 2:**
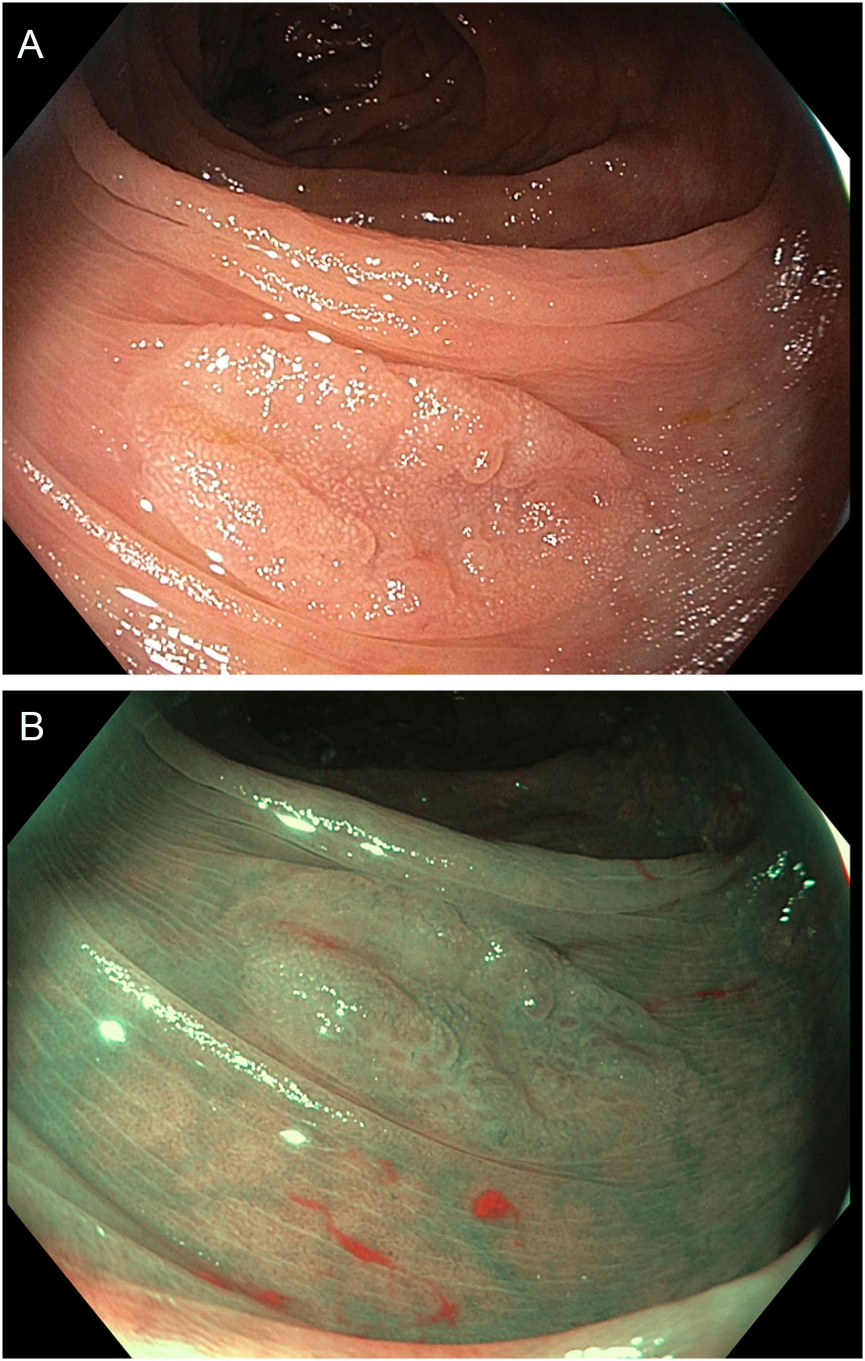
**A** and **B**: Colonoscopy showing a sessile serrated polyp with high-definition white light (**A**) and narrow band imaging (NBI) (**B**)

Germline genetic testing was performed with a gene panel consisting of 46 genes related to hereditary cancer and a nonsense variant in *RNF43* (c.1948 C > T, p.(Arg650Ter)) was identified. The variant had not previously been reported in gnomAD and was classified as likely pathogenic (class 4) according to ACMG guidelines. No other PVs were found. No other LPV or PV were identified (including the MMR genes). There was no family history of CRC or SPS, but information on family health related issues was sparse (See Fig. 2).

#### Family III

The patient, a 32-year-old Caucasian woman, underwent genetic counseling because of breast cancer (BC) in her mother at age 54. A paternal aunt suffered from an unknown cancer, referred to by the patient as probable cancer of the stomach, at the approximate age of 55. The proband was healthy. Germline genetic testing identified a nonsense variant in *RNF43* (c.1948 C > T, p.(Arg650Ter)). The variant had not previously been seen in gnomAD and was classified as likely pathogenic (class 4) according to ACMG guidelines. Following the genetic result, the proband underwent colonoscopy, which revealed one tubular adenoma in the sigmoid colon.

#### Family IV

A Caucasian woman aged 54 underwent genetic counseling because of BC in her mother and maternal grandmother. The proband never had cancer. She had previously undergone multiple colonoscopies due to Inflammatory Bowel Disease (IBD), the latest being 5 years prior to genetic counseling (no polyps detected). Germline genetic testing identified a nonsense variant in *RNF43* (c.1009 C > T, p.(Arg337Ter)). The variant was classified as likely pathogenic (class 4) according to ACMG guidelines. A subsequent colonoscopy identified no polyps.

## Discussion

We report four novel families with LPVs in *RNF43*. Our findings suggest variable penetrance of SPS. It is notable, that only three out of eight carriers (all relatives) had either serrated polyps or CRC. Of the variants none had previously been reported in literature in association with SPS. One of the identified variants occurred in two families.

Our findings demonstrate that not all carriers of LPV in *RNF43* fulfill the WHO criteria for SPS. Only two members of one of our families fulfilled the WHO criteria (2019) for SPS despite having undergone a colonoscopy at a relatively high age. In the previously published cases of patients with PV/LPV in *RNF43*, the patients were diagnosed with SPS between the age of 18‒65 (Supplementary Table 1). Additionally, we found one case of gastric cancer but no other extracolonic cancer corresponding to findings in previous studies [13, 14].

We acknowledge that the classification of variants in *RNF43* according to the general ACMG guidelines is a stretch, as *RNF43* could be considered a gene of unknown significance (GUS) and Mendelian inheritance is not firmly established. However, we have used these guidelines precisely to highlight the issue in classifying variants where a gene-disease link and Mendelian inheritance have not been firmly established. In 2 patients (in family 2 and 3) we identified the variant *RNF43*, c.1948 C > T, p.(Arg650Ter), which have not previously been linked to SPS. The variant is classified as likely pathogenic because it in theory results in nonsense mediated decay (NMD). However, the effects of the variant have not been investigated experimentally, and other genes have been found to escape NMD, thus, any disease association is still not proven.

The results from previous studies suggest some degree of ascertainment bias as most patients underwent genetic testing due to a phenotype suggestive of SPS or a family history thereof. Thus, the risk of being a carrier of a PV/LPV in *RNF43* and the associated increased risk of SPS/CRC in naive families is unknown.

Based on our findings, we find it speculative if PVs in *RNF43* should be regarded as a contributing polygenetic risk factor, more than a monogenetic cause. On the other hand, we do know that other cancer predisposition syndromes express varying degrees of decreased penetrance (*MSH6*,* PMS2*,* DICER1* etc.), thus a monogenic association is difficult to rule out before more carriers are identified and published. A family published by Chan et al., could support variable penetrance with only one patient with SPS in a large family with many carriers of the family variant in *RNF43* [15]. This highlights the complexity of genetic counseling in *RNF43* positive families, and whether healthy relatives should be offered predictive testing. This is a well-known issue in the field of hereditary cancer, where the indication and effect of surveillance programs are controversial ‒ to name a few: *CHEK2* and *ATM.* Although some guidelines have proposed surveillance in families that carry a LPV/PV in *RNF43*, there is a definite risk of overtreatment [16]. Thus, it seems reasonable to rely on the patient phenotype and whether the families fulfill the clinical criteria of SPS and not on the genetic findings alone. This is in accordance with recommendations from the American Consortia Guidelines which conclude that there are insufficient data to support multigene panel testing in patients with SPS without a family history of CRC and/or adenomatous polyposis [17]. British guidelines state that *RNF43* can be included in a gene panel for SPS, but that a routine recommendation is not warranted due to the low frequency of LPV/PVs in *RNF43* [18]. Thus, it should be considered whether *RNF43* should be included in multigene cancer panels, and if LPVs/PVs in *RNF43* should lead to colonoscopy surveillance in SPS and CRC naïve families.

In conclusion, we present four novel families with LPVs in *RNF43–* three of these without serrated polyps and/or CRC. Our results suggest that the rationale for integration of *RNF43* in broader cancer gene panels can be questioned– and that the genetic counseling of the family should be handled carefully, considering cancer and SPS family history and with information about reservations. Further research is needed to elucidate the role of *RNF43* in the risk of SPS and CRC.

## Electronic supplementary material

Below is the link to the electronic supplementary material.

Supplementary Material 1

Supplementary Material 2

## Data Availability

No datasets were generated or analysed during the current study. Supplementary material contains correspondance with the reviewers and the editors.
