# Supplementary material for: Germline pathogenic variants in RNF43 in patients with and without serrated polyposis syndrome
Source: Fam Cancer. 2024 Nov 15;24(1):3. doi: 10.1007/s10689-024-00428-6 (PMC11568000; doi:10.1007/s10689-024-00428-6)
Supplement: Supplementary file 1 — Supplementary Material 1 [file 10689_2024_428_MOESM1_ESM.docx]

Copenhagen, Denmark, July 2024

**To the Editor, *Familial Cancer***

On behalf of all authors, I’m pleased to submit this manuscript entitled: *Germline pathogenic variants in RNF43 in patients with and without Serrated Polyposis Syndrome***,** which we hope you will consider for publication.

Serrated Polyposis Syndrome is characterized by multiple and/or large serrated polyps in the colon and an increased risk of colorectal cancer, and it is one of the most frequent polyposis syndromes. Knowledge of the etiology is sparse, but for a small subset (fewer than 10 families) a monoallelic pathogenic variant in the gene *RNF43* have been found to cause serrated polyposis syndrome. Based on this *RNF43* is included in gene panels investigating a genetic predisposition to polyposis. However, the penetrance and phenotypic spectrum of patients with pathogenic variant in *RNF43* are poorly described.

In this short report we present four novel families with pathogenic variants in *RNF43*, three of them without any symptoms of serrated polyposis syndrome. Our results emphasize the difficulty in genetic counseling when detecting a variant in this gene, and that the gene should be integrated in gene panels with caution and excluded from broader cancer gene panels.

On behalf the authors, yours sincerely

**Anna Byrjalsen**

MD, PhD

Department of Clinical Genetics,

University Hospital of Copenhagen, Rigshospitalet

Denmark
