# Supplementary material for: Germline pathogenic variants in RNF43 in patients with and without serrated polyposis syndrome
Source: Fam Cancer. 2024 Nov 15;24(1):3. doi: 10.1007/s10689-024-00428-6 (PMC11568000; doi:10.1007/s10689-024-00428-6)
Supplement: Supplementary file 2 — Supplementary Material 2 [file 10689_2024_428_MOESM2_ESM.docx]

Regarding manuscript submission ID 2a74d158-2d0a-4929-895b-a83d5d2c40d1

Dear Editor, *Familial Cancer*

Firstly, we would like to thank you for your attention to our manuscript – and to reviewer 1 for assessing it. We know that it is a time-consuming task and we appreciate the effort.

We would, however, like to question the decision to reject the manuscript based on the following points:

1) Concerning classification of the *RNF43*, c.1948C>T, p.(Arg650Ter) reviewer 1 refers to the classification provided in ClinVar. The concerns we can read (from Invitae) in ClinVar does not point to classification, but rather whether the link between *RNF43* and polyposis is strong enough to be considered monogenetic. The aim of this publication is precisely to question whether this is the case, and why we think this publication is crucial to publish. And: if reviewer 1 accepts pathogenicity of other variants in this gene, we do not think he can use the argument reported in ClinVar, as they are based on whether there is a link between the gene (*RNF43*) and polyposis.

- We have made changes in the following places in the manuscript: p.3, l.50, p.4, l.81-82, p.6, l.148-149, p.7, l.179-181, and p.7. l.185-186.

2) Concerning the question of NMD, this is expected to occur, as there are more than 50 nucleotides to the exon junction (Lindeboom et al. The rules and impact of nonsense-mediated mRNA decay in human cancers, *Nat Genet*., PMID: 27618451, and Supek et al. To NMD or Not To NMD: Nonsense-Mediated mRNA Decay in Cancer and Other Genetic Diseases, *Trends Genet*., PMID: 33277042). Based on this it does not make sense to do functional analyses.

- We have included a sentence on using non-gene specific ACMG guidelines (p.4. l.97-99).
- And touched upon the same issue in the Discussion (p.6, l.158-162).
- We do not think it relevant to go further into functional testing of this variant, both considering the uncertainty of linkage between RNF43 and polyposis and due to the basic biology of NMD as stated above.

We appreciate the attention to the manuscript and now also to our rebuttal, and we urge you to reconsider publication of the manuscript. We will of course use the comments provided, to improve the manuscript before publication.

The absolute best wishes,

Anna Byrjalsen and Anne Marie Jelsig
